# Disclosure of herbal medicine use to health care providers among pregnant women in Nepal: a cross-sectional study

**DOI:** 10.1186/s12906-020-03142-9

**Published:** 2020-11-10

**Authors:** Mansoor Ahmed, Hyea Bin Im, Jung Hye Hwang, Dongwoon Han

**Affiliations:** 1 School of Public Health, Dow University of Health Sciences, Karachi, Pakistan; 2 Department of Global Health and Development, Graduate School, Hanyang University, Seoul, South Korea; 3 Institute of Health Services Management, Hanyang University, Seoul, South Korea; 4 Department of Obstetrics and Gynecology, Hanyang University College of Medicine, Seoul, South Korea; 5 Department of Preventive Medicine, Hanyang University College of Medicine, 222 Wangsimni-ro, Seongdong-gu, Seoul, 133-791 South Korea

**Keywords:** Physician-patient communication, Patient disclosure, Herbal medicine, Pregnancy, Nepal

## Abstract

**Background:**

Pregnant women’s disclosure of herbal medicine (HM) use to their health care providers during pregnancy is crucial, as misuse of HM can have a detrimental effect on both pregnant woman and the fetus. However, the lack of disclosure of HM use to physicians remains a public health concern in developing countries such as Nepal.

**Methods:**

A cross-sectional study was conducted among 400 postpartum women admitted at Maternity and Women’s Hospital located in Kathmandu, Nepal. The survey instrument included 30 questions on the use of HM during pregnancy, sociodemographic and health characteristics, and pregnancy outcomes. Chi-square test and logistic regression were conducted for data analysis using *S*PSS ver. 21.0., and a *p*-value of less than 0.05 was considered statistically significant for all analyses.

**Results:**

60.3% of respondents used at least one herbal remedy during their previous pregnancy, and the overall disclosure rate of HM use to healthcare providers was 54.6%. Women with secondary education level and four or more antenatal care visits were more likely to disclose their HM use to healthcare providers.

**Conclusions:**

This study highlights that despite the popular use of HM among pregnant women in Nepal, most women obtained HM-related information from informal sources and did not disclose their HM use to physicians. To ensure the safe use of HM, physicians should integrate questions regarding patients’ HM use into their routine patient assessments to facilitate active communication and improve the quality of care.

**Supplementary Information:**

The online version contains supplementary material available at 10.1186/s12906-020-03142-9.

## Background

The World Health Organization defines herbal medicines (HM) as various forms of herbal products and preparations containing active ingredients of plants [1]. Globally, the practice of HM use is widespread among pregnant women to treat common health problems for various reasons, such as cultural influences, easy accessibility, and cost-effectiveness of herbal products [2, 3]. The prevalence of HM use, which ranges from 1 to 63%, is notably greater in countries like Taiwan and Hong Kong, where traditional medicine use is widely accepted [3, 4]. However, misuse of HM during pregnancy remains a public health concern as pregnant women are more vulnerable to various physical and mental health issues [5–7]. The pharmacological characteristics of certain herbs can have a detrimental effect on both pregnant woman and the fetus [5, 8], and it was found that HM use is often self-prescribed and is unsupervised by health professionals [6, 7, 9].

One of the ways to prevent inappropriate use of HM is to increase patient disclosure of HM use to physicians, on the premise that the physicians have appropriate knowledge of herbs’ activity. It is because improved physician-patient communication on HM use provides an opportunity for appropriate medical intervention when complications or adverse reactions arise from concurrent use of HM and conventional medicine [10, 11]. Despite the importance of transparent communication between physicians and patients, previous research has found that 33 to 95% of pregnant women did not tell their healthcare providers about HM use simply because they were not asked or concerned about negative attitudes of medical doctors on HM use [3, 4, 6].

Previous studies have identified various socio-economic characteristics and healthcare utilization patterns to be associated with disclosure of HM use, and specifically, increased use of medical services was found to be associated with increased patient disclosure of HM use to healthcare providers [11]. Similarly, attending regular antenatal checkups (ANC) during pregnancy not only provides the healthcare providers to treat and monitor potential health problems, but also allows pregnant women to address concerns and questions about self-care activities during pregnancy, such as HM use [12, 13]. However, the potential association between frequent physician visits and disclosure of HM use among pregnant women has not been fully explored.

In Nepal, the use of HM is also widespread and is culturally embedded [14], yet little is known about the pattern of HM use during pregnancy, whether proper physician-patient communication on HM use has been practiced, and which factors are associated with disclosure of HM use to healthcare providers [14–16]. Therefore, this study aims to explore the pattern of HM use among pregnant women in Nepal, to examine the prevalence of disclosure of HM use during pregnancy, and to identify the factors associated with the disclosure of HM use to physicians during pregnancy.

## Methods

### Study setting and participants

A descriptive cross-sectional study was conducted among postpartum women admitted at Maternity and Women’s Hospital located in Kathmandu, Nepal. All postpartum women between the ages of 16 and 49 were invited to participate in the survey, and the survey was taken before they were discharged from the hospital to minimize the recall bias. Pregnant women with any type of disabilities or who were currently receiving treatment for a severe condition at a high dependency or an intensive care unit were excluded from participation.

### Study size

The sample size was calculated based on the formula for sample size determination using confidence interval (CI) of proportion: $n={z}_{\alpha /2}^2\bullet \frac{pq}{d^2}$. The margin of error was set at 0.05 with 95% confidence interval. Additionally, because there was no previous study on the use of HM among pregnant women in Nepal, a study from Malaysia was adopted as a reference for the estimated proportion of HM use during pregnancy (0.524) [17]. This equation gave us the minimum sample size of 383, and a total of 460 participants were invited to join the study to account for a 20% non-response rate.

### Survey instrument

After reviewing the literature [6, 18, 19], the semi-structured survey questionnaire was developed in English and then translated into the Nepali language to suit the target population. The Nepali questionnaire was back-translated into English to confirm the linguistic validity. In order to assess the content and face validity of the survey instrument, the questionnaire was reviewed by two physicians working in the obstetrics department at the study site and a healthcare practitioner at a local traditional medicine clinic for clarity, relevance, appropriateness, adequacy, and organization of the questions. The questionnaire was also pilot-tested on a sample of 20 participants to examine the length, clarity, and difficulty of the questions, and based on the results, a few items were modified into its final version.

The final version of the questionnaire contained 30 items with a combination of multiple-choice and open-ended questions (See Additional file 1: Survey Questionnaire). The survey was divided into four major sections. The first part of the questionnaire included seven questions regarding the medical characteristics of participants, such as current health-status measured in 5-point Likert scale, type of delivery, obstetric history, smoking status, number of antenatal care visits, complications experienced during pregnancy, labor, and after birth. The second part of the survey contained 12 questions concerning HM use during pregnancy (i.e., history of HM use before pregnancy, use of HM during pregnancy, types of modalities used and their indications, the reason for use and non-use, frequency of HM use, level of satisfaction, the experience of adverse effects, source of information, and disclosure of HM use to physicians). The third section comprised of five questions on characteristics of the newborn baby (i.e., gestational age at birth, gender, weight, congenital malformations, and neonatal symptoms experienced such as breathing problems, newborn jaundice, tender abdomen, temperature problems, bowel problems, lethargy, cardiac symptoms, and convulsions). The final section included six items on sociodemographic characteristics of respondents such as age, area of residence, education level, employment status, monthly household income, and traveling time from the nearest health facility.

### Data collection

The face-to-face interviews were conducted from July 4th, 2017 to August 12th, 2017. One supervisor and four data collectors were recruited for data collection. All participation was voluntary, and IRB-approved informed consent was obtained before the survey. In addition, information on complications during labor and after childbirth, as well as the characteristics of the newborn baby were obtained with the help of hospital staff. A total of 460 women were invited to participate in the study, and 400 completed the survey (87% response rate).

### Statistical methods

Although data from 400 participants were obtained, the current study focuses on disclosure of HM use to physicians during pregnancy, rather than general information around HM use among pregnant women. Therefore, only the data from 241 HM users were included in the final analysis. The data of 241 HM users were analyzed using the Statistical Package for Social Sciences (SPSS) version 21.0. Descriptive statistics were computed to examine sociodemographic and HM use-related characteristics of the respondents. Quantitative variables were grouped as follows: respondent’s age was grouped by 10-year increments (i.e., “less than 20 years old,” “between 21 and 30,” and “more than 31 years old”), and newborn baby’s birth weight was grouped by 5.0-kg increments starting from 2.5 kg (i.e., “less than 2.5kg,” “between 2.5 and 3.0kg,” and “more than 3.0kg”), as birth weight less than 2.5 kg are considered low birth weight. Pearson’s Chi-square test was performed to identify differences in sociodemographic and medical characteristics between those who revealed HM use to physicians during pregnancy and those who did not. Furthermore, variables that were statistically significant in the Chi-square test were included in regression for further analysis. The multivariate logistic regression analysis was conducted to determine factors associated with disclosure of HM use to physicians during pregnancy. A *p*-value of less than 0.05 was considered statistically significant for all analyses.

### Ethical clearance

The ethical approval of the study was given by the Institutional Review Board on Human Subjects Research and Ethics Committees, Hanyang University, Seoul, Korea (HYI-17-067-2). Additionally, formal permission to carry out the study was granted by the ethical body of the Maternity and Women’s Hospital in Kathmandu, Nepal (58-11 ka-259).

## Results

### Sociodemographic and pregnancy-related characteristics of study participants

Sociodemographic and pregnancy-related characteristics of respondents are presented in Table 1. The majority of respondents were between 21 and 30 years old (70.5%), received primary education or no education (52.3%), and were not employed (81.7%). As for pregnancy-related characteristics, most respondents experienced full-term pregnancy (91.3%), had normal delivery (62.2%), attended ANC four or more times (87.6%), and did not experience any complications during pregnancy (64.3%) or childbirth (58.1%).

**Table 1:** Sociodemographic characteristics of respondents

Characteristics	Total ***n*** = 241 (%)	Disclosed HM use ***n*** = 132 (%)	Did not disclose ***n*** = 109 (%)	***P***-value
**Age (years)**				
< 20	43 (17.8)	19 (14.4)	24 (22.0)	0.220
21–30	170 (70.5)	99 (75.0)	71 (65.1)	
≥ 31	28 (11.6)	14 (10.6)	14 (12.8)	
**Education level**				
Primary and under	126 (52.3)	54 (40.9)	72 (66.1)	< 0.001
Secondary	77 (32.0)	54 (40.9)	23 (21.1)	
College or above	38 (15.8)	24 (18.2)	14 (12.8)	
**Employment status**				
Yes	44 (18.3)	32 (24.2)	12 (11.0)	0.008
No	197 (81.7)	100 (75.8)	97 (89.0)	
**Household income**				
Low	72 (29.9)	30 (22.7)	42 (38.5)	0.015
Middle	52 (21.6)	28 (21.2)	24 (22.0)	
High	117 (48.5)	74 (56.1)	43 (39.4)	
**Residence**				
Rural	115 (47.7)	49 (37.1)	66 (60.6)	< 0.001
Urban	126 (52.3)	83 (62.9)	43 (39.4)	
**Perceived health status**				
Fair/worse	66 (27.4)	30 (22.7)	36 (33.0)	0.074
Good	175 (72.6)	102 (77.3)	73 (67.0)	
**Pregnancy term**				
Pre term	21 (8.7)	10 (7.6)	11 (10.1)	0.491
Full term	220 (91.3)	122 (92.4)	98 (89.9)	
**Type of delivery**				
Normal	150 (62.2)	90 (68.2)	60 (55.0)	0.036
Caesarian	91 (37.8)	42 (31.8)	49 (45.0)	
**ANC visits**				
≤ 3 times	30 (12.4)	7 (5.3)	23 (21.1)	< 0.001
4 or more	211 (87.6)	125 (94.7)	86 (78.9)	
**Birth weight**				
< 2.5 kg	39 (16.2)	19 (14.4)	20 (18.3)	0.249
2.5–3.0 kg	111 (46.1)	57 (43.2)	54 (49.5)	
> 3.0 kg	91 (37.8)	56 (42.4)	35 (32.1)	
**Complications during pregnancy**				
Yes	86 (35.7)	43 (32.6)	43 (39.4)	0.268
No	155 (64.3)	89 (67.4)	66 (60.6)	
**Complications during and after child birth**ª				
Yes	101 (41.9)	43 (32.6)	58 (53.2)	0.001
No	140 (58.1)	89 (67.4)	51 (46.8)	
Newborn complications*				
Yes	35 (14.5)	13 (9.8)	22 (20.2)	0.023
No	206 (85.5)	119 (90.2)	87 (79.8)	
**Congenital malformations/ birth defects on newborn**				
Yes	7 (2.9)	2 (1.5)	5 (4.6)	0.158
No	234 (97.1)	130 (98.5)	104 (95.4)	

ªProlonged labor, fetal distress, abnormal presentation, hypertension, postpartum hemorrhage, etc.

*Lethargy, breathing problems, fever, neonatal jaundice, etc.

54.8% of respondents disclosed HM use to healthcare professionals. Significant differences between the group who disclosed HM use to physicians and those who did not disclose HM use were observed in their education level (*p* < 0.001), employment status (*p* = 0.008), household income (*p* = 0.015), residential area (*p* < 0.001), type of delivery (*p* = 0.036), number of ANC visits (*p* < 0.001), the experience of complications during and after childbirth (*p* = 0.001), and experience of newborn complications (*p* = 0.023).

### Types of HM used during pregnancy and reported indications

Table 2 shows the types of HM modalities used by respondents during pregnancy. Consumption of ginger (46.1%) and turmeric (43.2%) were the highest, followed by lemon tea (29.5%), tulsi (24.5%), and olive oil (12.0%). The most commonly reported indications for HM use (not shown in Tables) were cough, cold, and flu (72.6%), nausea and vomiting (39.8%), and heartburn and indigestion (23.6%).

**Table 2:** Types of HM modalities used during pregnancy

HM modalities	***n*** = 241 (%)*	Reported indications	n (%)*
Ginger	111 (46.1)	Cold/flu	86 (77.5)
		Cough	84 (75.7)
		Nausea/vomiting	44 (39.6)
		Heartburn	23 (20.7)
Tumeric	104 (43.2)	Cold/flu	98 (94.2)
		Cough	66 (63.5)
		Skin condition	8 (7.7)
		Inflammation	3 (2.9)
Lemon tea	71 (29.5)	Nausea/vomiting	66 (93)
		Heartburn	26 (36.6)
		Cough	19 (26.8)
		Headache	3 (4.2)
Tulsi	59 (24.5)	Cough	53 (89.8)
		Heartburn	33 (55.9)
		Improve immunity	2 (3.4)
		Hypertension	1 (1.7)
Olive oil	29 (12.0)	Massage	26 (89.6)
		Skin condition	13 (44.8)
Neem	25 (10.4)	Skin condition	15 (60.0)
		Hypertension	7 (28.0)
		Fever	3 (12.0)
		Stomach upset	1 (4.0)
Garlic	21 (8.7)	Abdominal pain	19 (90.5)
		Cold/flu	7 (33.3)
		Fatigue	1 (4.8)
		Hypertension	1 (4.8)
*Aloe vera*	18 (7.5)	Skin/hair	16 (88.9)
		Constipation	6 (33.3)
		Stomach upset	4 (22.2)
		Hypertension	1 (5.6)
Peppermint	12 (5.0)	Cold/flu	7 (58.3)
		Abdominal pain	6 (50.0)
		Heartburn	3 (25.0)
		Nausea/vomiting	1 (8.3)
Mustard oil	10 (4.1)	Relaxant	9 (90.0)
		Fatigue	1 (10.0)
		Cold/flu	1 (10.0)
Rice water	4 (1.7)	Burning micturition	4 (100.0)
Bitter gourd juice	4 (1.7)	Hypertension	4 (100.0)
Cumin seed/powder	2 (0.8)	Stomach upset	1 (50.0)
		Fever with headache	1 (50.0)
Green tea	1 (0.4)	Hypertension and headache	1 (100.0)

*Columns do not add up to 100% due to the selection of multiple answers

### Source of information on HM

As presented in Table 3, the primary source of information regarding HM use was family, friends, or neighbors (98.3%) and newspapers or magazines (17.1%). 4.6% of respondents obtained HM information from doctors.

**Table 3:** Source of information on HM use

Source of information (***n*** = 241)	n (%)*
Family/friends/neighbor	236 (98.3)
Newspaper/magazine	41 (17.1)
Doctor	11 (4.6)
Herbalist	5 (2.1)
TV/radio/internet	4 (1.7)
Midwife/health worker	2 (0.8)
Temple/religious text	1 (0.4)
Self	1 (0.4)

*Columns do not add up to 100% due to the selection of multiple answers

### Reasons for HM use and non-disclosure of HM use to physicians

The most common reason for HM use among pregnant women was due to its perceived safety (66.4%), followed by family tradition and culture (63.1%) and perceived-effectiveness in treating their conditions (48.1%). Regarding the reason for non-disclosure of HM use to healthcare providers, 39.8% responded that doctors did not ask, and 29.6% reported that they did not think it was important to tell the doctors about their use (Table 4).

**Table 4:** Reason for HM use and non-disclosure

**Reason for HM use (*n*** **= 241)**	n (%)*
I believe it is safe	160 (66.4)
Family tradition/culture	152 (63.1)
I believe it is effective	116 (48.1)
It is cheap and accessible	25 (10.4)
**Reason for non-disclose (*n*** **= 109)**	**n (%)**
Doctors did not ask	43 (39.8)
It was not important	32 (29.6)
Should have informed but forgot	19 (17.6)
Afraid of doctors’ response	12 (11.1)
No ANC visit	2 (1.9)

*Columns do not add up to 100% due to the selection of multiple answers

### Factors associated with disclosure of HM use during pregnancy

The result of multivariate logistic regression analysis is shown in Table 5. Having secondary education level (OR: 2.25, CI: 1.12–4.51) and attending ANC four or more times (OR: 3.74, CI: 1.41–9.91) were associated with disclosure of HM use to healthcare professionals.

**Table 5:** Multivariate logistic regression analysis between demographic and pregnancy-related factors and physician disclosure on HM use

Characteristics	OR	95% CI	***P***-value
**Education level**			
Primary and under	1	Ref	
Secondary	2.255	1.127–4.513	0.022
College or above	1.575	0.656–3.783	0.310
**Household income**			
Low	1	Ref	
Middle	1.072	0.479–2.401	0.865
High	1.362	0.668–2.780	0.395
**Employment status**			
Yes	1	Ref	
No	0.883	0.376–2.073	0.774
**Residence**			
Rural	1	Ref	
Urban	1.807	0.985–3.315	0.056
**ANC visits**			
≤ 3 times	1	Ref	
4 or more	3.747	1.416–9.915	0.008
**Type of delivery**			
Normal	1	Ref	
Caesarian	0.556	0.307–1.009	0.054
**Complications during and after child birth**ª			
Yes	1	Ref	
No	1.596	0.890–2.862	0.117
**Newborn complications**			
Yes	1	Ref	
No	2.183	0.962–4.953	0.062

## Discussion

This cross-sectional study examined the characteristics and potential predictors of HM use among pregnant women in Nepal. Our results revealed that 60.3% of Nepali women used at least one herbal remedy during their previous pregnancy, which was higher compared to the prevalence found in more developed countries [3]. Ginger (46.1%), turmeric (43.2%), and lemon tea (29.5%) were the most frequently used HM among Nepali women. While the use of ginger was well documented in previous literature [20, 21], several studies have also found a high prevalence of raspberry leaf, Chinese herbs, Echinacea, and Chamomile uptake [20–24]. These differences in the prevalence and types of HM use can be attributed to various socio-demographic and cultural characteristics.

Comparable to previous findings, cold and flu-like symptoms and nausea/vomiting were the most frequently reported indications for HM use [2, 24, 25]. Ginger was the most commonly used HM modalities to manage those conditions, and its use is prevalent in both pregnant women and the general population [26]. In fact, several systematic reviews indicate that ginger is an effective and safe treatment for pregnancy-induced nausea and vomiting [27–29]. For common cold symptoms, the second most commonly used modality was turmeric, and in Nepal, turmeric powder is usually stirred with hot milk and ingested to treat common cold [30].

However, the consumption of certain herbal remedies without appropriate knowledge can have detrimental effects on the health of the pregnant mother and fetus [5, 31]. For instance, the ingestion of turmeric in pregnancy can stimulate uterine contractions and cause abortion [32]. Basil should be used in pregnancy with caution and under the supervision of a qualified healthcare practitioner [33]. Minor malformations were observed after high doses of neem in pregnant rats, whereas safety information in human pregnancy has not been established yet [34].

Nevertheless, the most frequently reported source of information for HM use during pregnancy in this study were family, friends, and neighbors (98.3%), and only 7.5% of respondents obtained the information from health professionals such as doctors, herbalists, midwives, and health workers. Although the credibility of information provided by non-healthcare professionals cannot be guaranteed [35], such dependency on informal sources of information was also seen in other studies [3, 21]. Consistent with previous studies, these findings reflect the need for healthcare professionals to have an adequate understanding of HM use during pregnancy to ensure the safety of mother and child and prevent potential misuse.

As such, despite the high utilization and lack of adequate source of information on HM, only 54.8% of respondents have disclosed HM use to healthcare professionals during pregnancy. Although the disclosure rate among Nepali women was higher compared to other studies [4, 21, 36, 37], almost half of the respondents failed to communicate with their physicians on HM use, and several studies revealed the negative consequences of poor communication between patients and healthcare professionals on HM use [35, 38]. Furthermore, consistent with previous findings, commonly reported reasons for non-disclosure included doctors not asking about HM use, and pregnant women’s belief that it is not important to disclose HM use [10, 39]. These reasons can be attributed to family and cultural influences on HM use. Depending on socio-cultural backgrounds, pregnant women may not consider HM as ‘medicines,’ and they may not think that it is necessary to disclose HM use to their physicians. This implies that in order to promote transparent communication and ensure proper use of HM among pregnant women, healthcare providers should actively initiate the conversation about the use of non-conventional remedies [10, 40]. Moreover, healthcare providers must have knowledge of herbal activities so that they may provide recommendations to promote the safe use of HM.

Women’s education level (*p* = 0.022) and the number of antenatal care (ANC) visits (*p* = 0.008) were identified as potential predictors of disclosure of HM use to physicians. Previous studies also found that higher educational attainment was associated with disclosure of non-conventional medicine use [41, 42]. These associations could be attributed to people with higher education levels having better health literacy, which may influence them to seek advice and information on HM use from healthcare providers [43, 44]. Furthermore, building mutual trust through regular antenatal care visits may have affected pregnant women to communicate with their physicians on HM use. Similarly, previous studies also found a positive correlation between the disclosure of non-conventional medicine use and frequent doctor visits and higher scores on the patient-provider relationship scale [11, 45].

The limitations of this study include recall bias and generalizability of the results, as the data was collected retrospectively at a single hospital in Kathmandu city. However, the hospital, where the study was conducted, is considered as the largest public tertiary maternity hospital in Nepal. Furthermore, in order to reduce recall bias, the survey was taken among postpartum women admitted to the inpatient unit before they were formally discharged from the facility.

## Conclusion

This study found that the use of HM during pregnancy was common in Nepal. However, most women obtained HM-related information from informal sources and did not disclose HM use to their physicians. ANC visits and women’s education levels were found to be associated with disclosure of HM use to physicians. Therefore, to provide optimal care and promote coordination between HM and conventional medicine, healthcare providers should stay up to date with the knowledge of HM use. Furthermore, integration of questions on HM use into routine patient assessments during ANC should be considered to facilitate active communication on HM use during pregnancy. Lastly, public education and awareness programs on the safe use of HM should be developed to encourage pregnant women’s disclosure of HM use.

## Supplementary Information

**Additional file 1.**

## Data Availability

The data will be accessible by contacting the corresponding author of this study.

## Abbreviations

HM: Herbal medicine
ANC: Antenatal care
