# Supplementary material for: Disclosure of herbal medicine use to health care providers among pregnant women in Nepal: a cross-sectional study
Source: BMC Complement Med Ther. 2020 Nov 10;20:339. doi: 10.1186/s12906-020-03142-9 (PMC7654584; doi:10.1186/s12906-020-03142-9)
Supplement: Supplementary file 1 — Additional file 1. [file 12906_2020_3142_MOESM1_ESM.pdf]

Questionnaire No..............   Name of the hospital...............................

Questionnaire on: Safety concerns regarding use of herbal medicine among pregnant women in selected Asian Countries

<u>To the participant involved in this study:</u>

We would highly appreciate you for agreeing to participate in this research study: "**Safety concerns regarding use of herbal modalities among pregnant women in selected Asian Countries**", and for spending your time to participate in this interview which will take around 10 -15 minutes. Your input in this study is important to help us in investigating the factors influencing the use of Herbal Medicine among pregnant women in Asian countries. The information gained from this questionnaire will be used for academic purposes only and to provide insight to recommend further interventions to improve the healthcare and health status of women during pregnancy.

Department of Preventive Medicine, Hanyang University

Research Supervisor – Professor: Dong Woon Han

Researcher – Mansoor Ahmed (mmahar85@outlook.com)

<u>Certificate of Consent</u>

I have been invited to participate in this survey. The foregoing information has been read to me. I have had the opportunity to ask questions about it and any questions I have asked have been answered to my satisfaction. I have noticed that my participation in this study is totally voluntary and I have the right to withdraw from the interview at any time without in any way affecting my medical care. I consent voluntarily to be a participant in this study.

Signature of respondent ______________________________

Section A: Questions on your medical characteristics: I would like to ask you a few questions about your health status. Please check (√) the appropriate responses.

1. In general, would you say your current health is:

☐①Excellent ☐②Good ☐③Fair ☐④Poor ☐⑤Very poor

2. Type of delivery of last pregnancy

☐①Normal ☐②Caesarian ☐③ Forceps ☐④Vacuum ☐⑤ Other ___________

3. Obstetric history (write appropriate numbers in each box)

3-1. Number of times you have become pregnant	3-2. Age at first pregnancy

4. Does anyone smoke cigarette or shisha/hookah in your home?

☐① Yes ☐② No

5. Number of times antenatal care services received during last pregnancy

☐① None ☐② 1 to 3 times ☐③ 4 or more times

6. Check (√) the complications, if you experienced/diagnosed, <u>during last pregnancy</u> (may select more than one) [See the pregnancy file if possible]

☐ 1) Preeclampsia/hypertension ☐ 2) Oligohydramnios
☐ 3) Polyhydramnios ☐ 4) Convulsions
☐ 5) Fainting ☐ 6) High grade fever
☐ 7) Urinary tract infection ☐ 8) Gestational diabetes
☐ 9) Severe anemia ☐ 10) Premature rupture of water bag
☐ 11) Severe headache ☐ 12) Hyperemesis gravidarum
☐ 13) Placenta previa ☐ 14) Placental abruption
☐ 15) Others ____________________ ☐ 16) None

7. Check (√) the complications, if you experienced/diagnosed, <u>during labor and/or after your last delivery</u> (may select more than one) [See the pregnancy file if possible]

☐ 1) Prolonged labor ☐ 2) Abnormal presentation
☐ 3) Fetal stress ☐ 4) Profuse bleeding
☐ 5) Blood transfusion ☐ 6) Urinary or fecal incontinence
☐ 7) Hypertension ☐ 8) Hemorrhoids

☐ 9) Severe anemia
☐ 10) Smelly vaginal discharge
☐ 11) Severe headache
☐ 12) High grade fever
☐ 13) Convulsions
☐ 14) Postpartum depression
☐ 15) Others ____________________
☐ 16) None

Section B: Use of Herbal Medicine during your most recent pregnancy

Explanation: Herbal Medicine means 'Any herb (such as ginger, tulsi, garlic, turmeric, lemon tea) or herbal preparation (syrup, paste, powder) used during your most recent pregnancy either self-prescribed by you or recommended by family/friends/other person or health professional in order to manage any symptoms, prepare for delivery or to support baby's development'.

8. Did you ever use any herb (such as ginger, tulsi, garlic, turmeric, lemon tea) or herbal preparation (syrup, paste, powder) to improve your health before your last pregnancy?

☐ ① Yes ☐ ② No

9. Did you use any herb (such as ginger, tulsi, garlic, turmeric, lemon tea) or herbal preparation (syrup, paste, powder) to improve your health during your last pregnancy?

☐ ① Yes ☐ ② No (go to Question 19)

10. Check (√) all of the following herbs that you used, for which symptoms, to manage your health, to prepare for labor and to support baby's development. (may select more than one)

Herbs	Indications (reason for use)			
1) Ginger (or its tea)	☐Cough	☐Cold/flu	☐Heartburn	☐Vomiting/nausea
2) Tulsi (holy basil)	☐Cough	☐Heartburn	☐Improve immunity	☐Hypertension
3) Garlic (lahsun)	☐Abd. pain	☐ Cold/flu	☐Fatigue	☐Hypertension
4) Turmeric (haldi)	☐Cough	☐Cold/flu	☐Skin condition	☐Anti-inflammatory
5) Lemon tea	☐Cough	☐Vomiting/ nausea	☐Heartburn	☐Other_______
6) Neem	☐Stomach upset	☐Skin condition	☐Hypertension	☐Other_______
7) Peppermint (pudeena or its tea)	☐Abd. pain	☐Cold/flu	☐Heartburn	☐Other_______

8) Olive oil	☐Skin condition	☐Massage	☐Nutrition	☐Other_______
9) Aloe vera	☐Skin/hair	☐Constipation	☐Stomach upset	☐Other_______
11) Other herbs ________________	☐ ___________	☐ ___________	☐ ___________	☐ ___________

11. Check (√) the symptoms or conditions for which you used herbal medicine, <u>during last pregnancy</u> (may select more than one)

☐ 1) Nausea/vomiting ☐ 2) Heartburn/indigestion
☐ 3) Cough/cold/flu ☐ 4) Fatigue
☐ 5) Back pain/ joint pain ☐ 6) Abdominal pain
☐ 7) To help normal delivery ☐ 8) Promote baby's development
☐ 9) Skin marks/striae gravidarum ☐ 10) Constipation
☐ 11) Anxiety/depression ☐ 12) Headache
☐ 13) Insomnia/sleeplessness ☐ 14) As relaxant
☐ 15) Others ___________________

12. How frequently did you use the above herbal medicine during pregnancy?

☐① Daily ☐②two or more times a week ☐③ Weekly

☐④ Occasionally ☐⑤ Only once

13. How satisfied were you with use of the above herbal medicine during pregnancy?

☐①Very satisfied
☐②Satisfied
☐③Undecided
☐④Unsatisfied
☐⑤Very unsatisfied

14. Did you experience any of the following effects after use of the above herb or herbal preparation during last pregnancy? (may select more than one)

☐ 1) Abdominal pain ☐ 2) Diarrhea
☐ 3) Constipation ☐ 4) Nausea/vomiting
☐ 5) Dry mouth ☐ 6) Urine problems
☐ 7) Skin rash ☐ 8) Severe headache
☐ 9) Fatigue ☐ 10) Dizziness/drowsiness
☐ 11) Sedation/sleepiness ☐ 12) Palpitation/hypertension
☐ 13) Edema ☐ 14) Allergic reaction

☐ 15) Loss of appetite ☐ 16) Other________________

☐ 17) None (go to Question 15)

14-1. Name of herb or herbal preparation that caused above effects: ___________________

14-2. What was the herb taken for? (indication) ____

14-3. Frequency: ☐①Daily ☐②Couple of times a week ☐③Occasionally ☐④Once

15. Who recommended herbal medicine you used during pregnancy (may select more than one)

☐ ① Family/friends/neighbor ☐ ② Herbalist ☐ ③ Doctor

☐ ④ Midwife or health worker ☐ ⑤ Newspaper/magazine ☐ ⑥ TV/radio/internet

☐ ⑦ Temple/religious text ☐ ⑧Other___________________

16. Did you inform your doctor or midwife about herbal medicine you used during pregnancy?

☐ ① No ☐ ② Yes (go to Question 18)

17. If no, why you didn't inform your doctor or midwife?

☐ ① Doctor didn't ask ☐ ② It was not important

☐ ③ Afraid of doctor's response ☐ ④ Should have informed but I forgot

☐ ⑤ Other_________________________________

18. What is the reason you used above herbal medicine during pregnancy? (may select more than one)

☐①I believe it's effective ☐②I believe it's safe ☐③Family, tradition or culture

☐④ It's cheap and accessible ☐⑤I am not satisfied with modern medicine

19. What is the reason you did not use above herbal medicine during pregnancy? (may select more than one)

☐①It's not effective ☐②It's not safe ☐③I am satisfied with modern medicine

☐④It's expensive and difficult to get ☐⑤My family didn't let me use ☐⑥My doctor/nurse didn't let me use

Section C: Characteristics of the Newborn baby **[see the newborn card if possible]**

20. Gestational age at birth (please write exact number in weeks) _________ weeks

21. Gender of the newborn

☐ ① Boy ☐ ② Girl

22. Weight of the newborn (please write exact number in grams) ______________ grams

23. Any congenital malformations/birth defects to the newborn? (may select more than one) [see the newborn card if possible]

☐ 1) Nervous system anomalies (anencephaly, hydrocephalus etc.)

☐ 2) Cleft lip/palate ☐ 3) Cardiac anomalies

☐ 4) Genital organ anomalies ☐ 5) Down Syndrome

☐ 6) Tongue/mouth/pharynx ☐ 7) Imperforate anus

☐ 8) Congenital malformations of ☐ 9) Other_________________

☐ 10) None

24. Check the symptoms if the newborn baby experienced (may select more than one) [see the newborn card if possible]

☐ 1) Breathing problems ☐ 2) Appearance (blue or pale)

☐ 3) Newborn jaundice (yellow skin, eyes) ☐ 4) Convulsions/unconsciousness

☐ 5) Excessively irritable and crying ☐ 6) Pus, bleeding around umbilicus

☐ 7) Tender or tense abdomen ☐ 8) Fever

☐ 9) Body gets cold easily ☐ 10) Any infections

☐ 11) Bowel problems (blood in bowel, diarrhea)

☐ 12) Activity (weak suck on feeding, lethargic, no muscle activity)

☐ 13) Cardiac problems (abnormal heart rate or blood pressure)

☐ 14) Others _______________________________ ☐ 15) None

Section D: Sociodemographic characteristics of the study participant

25. What is your age? completed years __________

26. Where do you live?

☐ ① Rural area ☐ ② Urban area

27. What is the highest level of education you have completed?

☐ ①No education (Illiterate) ☐ ②Elementary ☐ ③High school ☐ ④College/university

28. Are you employed?

☐ ①Yes ☐ ②No (housewife)

29. What is monthly income in your home?

☐ ①Less than Nrs. 10,000 ☐ ②Nrs. 10,001 to 20,000 ☐ ③Nrs. 20,001 to 30,000

☐ ④Nrs. 30,001 to 40,000 ☐ ⑤Nrs. 40,001 and above

30. Time to get to the nearest health facility

☐ ①Less than 30 minutes ☐ ②30 minutes to 1 hour ☐ ③More than 1 hour

---THANK YOU--
